# Improved real-time exercise stress cardiac cine imaging using self consistent parallel imaging with temporal sensitivity estimation (TSPIRIT)

**DOI:** 10.1186/1532-429X-14-S1-P254

**Published:** 2012-02-01

**Authors:** Hui Xue, Yu Ding, Ti-Chiun Chang, Christoph Guetter, Subha V Raman, Marie-Pierre Jolly, Orlando P Simonetti

**Affiliations:** 1 Siemens Corporate Research, Princeton, NJ, USA; 2 The Ohio State University, Dorothy M. Davis Heart and Lung Research Institute, Columbus, OH, USA

## Summary

To improve the image quality and increase the signal-noise-ratio of real-time exercise stress cardiac cine imaging, we extended the recently proposed SPIRIT image reconstruction by incorporating temporal coil sensitivity estimation and spatial regularization. The proposed method was tested on 10 volunteers and the average SNR gain was 38.2% without increasing ghosting artifacts.

## Background

Myocardial wall motion can now be assessed using CMR immediately following treadmill exercise [1,2]. Given that stress-induced wall motion abnormalities rapidly fade after cessation of exercise, imaging must be completed as quickly as possible. Additionally, shortness of breath following exercise precludes the use of segmented k-space acquisitions making real-time imaging the only practical choice for post-exercise cine. While this eliminates the needs for ECG triggering and breath-holding, signal-noise-ratio (SNR) and temporal and spatial resolution are typically sacrificed. Image degradation can be more severe for post-exercise cine due to rapid heart rate and exaggerated breathing. To improve the quality of exercise stress cine, we propose to extend the SPIRIT reconstruction technique [3] by incorporating temporal sensitivity estimation (TSPIRIT) and spatial regularization.

## Methods

10 healthy volunteers (6 males; age 23.1−41.1 yrs) with normal left ventricular thickness underwent free-breathing real-time exercise stress cine examinations after having given written consent. An MR compatible treadmill system was utilized [1] together with a 1.5T scanner (Avanto, Siemens) and a 32-channel coil (Rapid MRI). Three slices (one short-axis and two long-axis views) were acquired in each subject using the following sequence parameters: bSSFP, TR1.09/TE0.9ms, image matrix 160×80, flip angle 58°, resolution 2.44×2.44 mm^2^, bandwidth 1420Hz, acceleration rate 4 with time-interleaved sampling of k-space.

All temporally interleaved k-space frames were averaged to generate ACS lines and GRAPPA was first used to estimate full k-space for every frame. Karhunen-Loeve transform filtering was applied to improve sensitivity estimation, and SPIRIT calibration was performed for every frame. The under-sampled k-space and estimated kernels served as inputs to a non-linear solver. An LSQR matrix inversion solver was performed, and then a non-linear conjugate gradient solver was called with spatial regularization. Final images were generated after convergence and compared to images of conventional TGRAPPA reconstruction [4] of the same raw data. SNR was estimated [5] and ghosting artifacts caused by chest wall motion were quantified by computing the peak spatial cross-correlation ratio along the phase-encoding direction [6].

## Results

**Figure 1:**
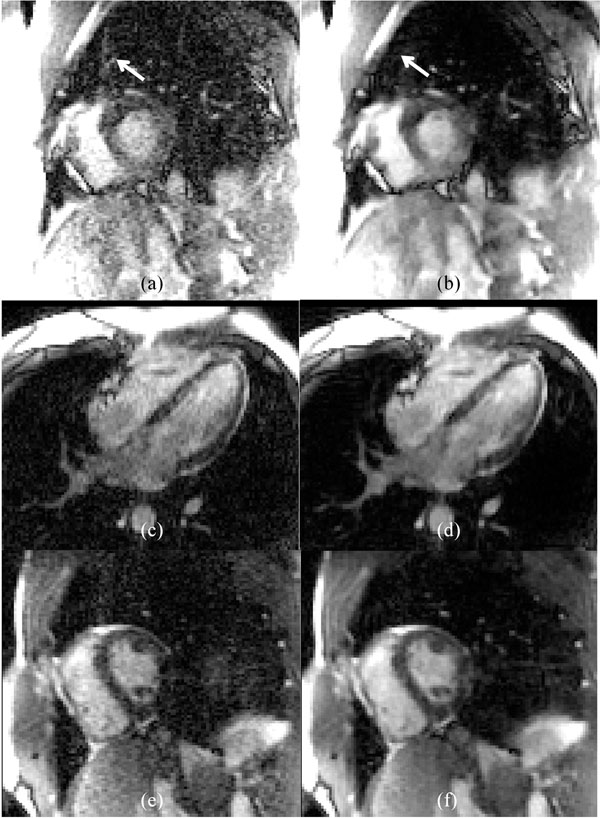
Example images acquired using acceleration rate 4 and reconstructed using linear TGRAPPA (a,c,e) and non-linear TSPIRIT (b,d,f). Note increase in SNR with TSPIRIT and reduced ghost artifact (white arrows in a and b). The non-linear nature of TSPIRIT could lead to more favorable trade-off between SNR and artifacts, compared to the linear nature of TGRAPPA.

**Figure 2:**
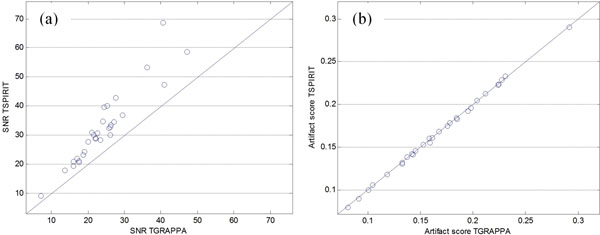
Plots of measured SNR (a) and artifact scores (b) for TSPIRIT and TGRAPPA. The SNR gain is significant for all cases while artifacts remain on the same level as the TGRAPPA. Mean SNR/artifact scores 32.3±12.6/0.169±0.049 for TSPIRIT and 24.1±8.44/0.169±0.048 for TGRAPPA.

Figure 1 shows an example of the improved image quality of the proposed TSPIRIT technique compared to TGRAPPA. Figure 2 plots measured SNR and artifact scores, showing a significant increase of SNR (mean relative gain 38.2±17.8%, P<1e-5) without raising ghosting artifacts (P=0.960). This is consistent with visual reading.

## Conclusions

A new TSPRIT reconstruction scheme has been proposed by extending the SPIRIT method with temporal sensitivity estimation and spatial regularization. This technique was shown to significantly improve the quality of exercise stress real-time cine imaging. In vivo testing shows 38.2% SNR gain can be achieved without raising ghosting artifacts.

## Funding

NIH grant R01 HL102450
